# Associations between cigarette prices and consumption in Europe 2004–2014

**DOI:** 10.1136/tobaccocontrol-2019-055299

**Published:** 2020-06-16

**Authors:** Anthony A Laverty, Christopher Millett, Filippos T Filippidis

**Affiliations:** Public health policy evaluation unit, School of Public Health, Imperial College London, London, London, UK

**Keywords:** global health, price, tobacco industry

## Abstract

**Introduction:**

Increases in tobacco price are known to reduce smoking prevalence, but these correlations may be blunted by the availability of budget cigarettes, promoted by the tobacco industry to maintain profits.

**Objective:**

To investigate the effect of budget cigarettes on cigarette consumption using data from Europe 2004–2014.

**Methods:**

Data on the annual population-weighted cigarette consumption per adult come from the International Cigarette Consumption Database. Data on the annual tobacco price come from Euromonitor International for 23 European countries. Median prices and price differentials (operationalised as percentages obtained by dividing the difference between median and minimum prices by the median price) were examined. A linear random-effects model was used to assess associations between median prices and price differentials with cigarette consumption within 1 year and with a 1-year time lag.

**Results:**

Cigarette consumption per capita declined over the study period (−29.5 cigarettes per capita per year, 95% confidence interval −46.8 to −12.1). The analysis suggests that increases in cigarette price differentials, a marker of opportunities for smokers to switch to less expensive cigarettes, may be associated with greater consumption in the same year (6.4 for a 10% increase in differential, −40.0 to 52.6) and are associated with greater consumption in the following year (67.6, 25.8 to 109.5).

**Conclusion:**

These analyses suggest that even in Europe, where tobacco taxes are relatively high compared with other regions, differential cigarette pricing strategies may undermine tobacco control. Further research is needed on links between tobacco price structures and consumption, and policy design to maximise the effectiveness of tobacco taxation.

## Introduction

Recent analyses have cast doubt on the extent to which the Framework Convention on Tobacco Control (FCTC) is achieving its aim of reducing tobacco smoking.^1^ These analyses highlight differential success across regions and nations and serve as a timely reminder of the continued efforts of the tobacco industry to undermine tobacco control policies, which, when implemented properly, have been shown to be effective.^2 3^ One prominent approach of the tobacco industry is the use of pricing mechanisms which maintain the availability of budget cigarettes.^4^ Previous research has highlighted the important role of tobacco prices, but the tobacco industry has responded to increased taxation with a range of mechanisms to ensure the continued availability of budget cigarettes.^5–7^

Globally, pricing strategies are perhaps most important in low- and middle-income countries (LMICs), where cigarette price differentials are generally larger than in high-income countries.^8^ The European Union provides potentially important lessons with its policy on cigarette taxation and prices, which includes measures to counter industry's efforts to maintain and promote budget cigarettes. It has adopted a number of regional policies, including a minimum excise tax burden and an excise tax floor,^9^ which aims to reduce variation in tax policy between EU member states. These have resulted in overall increases of average prices, although differences in individual tax arrangements have meant that this has not been uniform across countries.^10 11^ We thus use data from Europe to assess links between cigarette prices, differences between average and budget cigarettes, and consumption.

## Methods

Using data on consumption and inflation-adjusted tobacco prices, we conducted a linear random-effects regression of the relation between price and consumption in 23 European countries from 2004 to 2014.

Our primary outcome is cigarette consumption per capita, from the International Cigarette Consumption Database (ICCD), which provides annual population-weighted cigarette consumption per adult.^12 13^ We used data on cigarette prices from the market research company Euromonitor International, which records cigarette pack prices in many countries annually, covering the market share of at least the top 10 brands in each country.^14^ We obtained data on 23 European countries (all EU member states except Austria, Croatia, Malta, Cyprus and Luxembourg). A median of 93 (IQR 58 to 184) cigarette products was sampled in each country each year. We also used annual data for each country on the unemployment rate among the population aged >15 years, extracted from Eurostat.^15^

We used the Harmonised Index of Consumer Prices to adjust all tobacco prices for inflation and transformed them into euros using the exchange rate on the 30th of June of the relevant year.^15^ We calculated minimum and median prices for 20 cigarettes (one pack). Our main explanatory variables were median prices and price differentials in each country. We calculated the price differential between minimum and median cigarette prices, which is expressed as a percentage of the median cigarette price (ie, price differential=100×(median cigarette price – minimum cigarette price)/median cigarette price)).

We fitted a linear panel regression model, with cigarette consumption per capita as the dependent variable. We compared fixed- and random-effects specifications using the Hausman test, which indicated that the random-effects model with generalised least squares estimator was appropriate. In addition to median price per pack and price differential, we used their respective 1-year lag terms as independent variables, to account for potential delays in associations between price and consumption.^14 16^ The model additionally controlled for unemployment levels of countries. Regression results are presented as β coefficients, representing the number of cigarettes consumed per capita annually, with the respective 95% confidence intervals.

To test the robustness of these results we also conducted analyses without including time lags for the price effects, and controlling for gross domestic product (GDP) per capita at the country level.

## Results

Median and minimum cigarette prices for all 23 countries have been reported in a previous paper using the same price data.^14^ Briefly, inflation-adjusted median price for 20 cigarettes across all 23 countries in 2004 was €2.16, which rose to €3.60 in 2014. Price differentials decreased over time, with the median price differential being 24.6% in 2004, decreasing to 12.8% in 2014. This means that in 2014, the least expensive cigarettes were 12.8% cheaper than the median priced cigarettes.

In the linear random-effects, panel regression model, cigarette consumption per capita declined over the study period (−29.5 cigarettes per capita, annually, 95% CI −46.8 to −12.1)(table 1). Country-level unemployment was negatively associated with the number of cigarettes consumed (−41.5 cigarettes per capita per 1% rise in unemployment, −53.0 to −30.15).

**Table 1:** Associations between median cigarette pack prices and price differentials with population-weighted cigarette consumption per capita in 23 European countries 2004–2014

	β (95% CI)
Median cigarette price (per €1 increase)	
Within year	−**113.0 (−225.7 to −0.2**)
1 year lag	−49.5 (−164.1 to 65.1)
Price differential between median and minimum cigarette price (per 10% increase)	
Within year	6.4 (−40.0 to 52.6)
1 year lag	**67.6 (25.8 to 109.5**)
Unemployment (per 1% increase)	−**41.5 (−53.0 to −30.1**)
Time (annual)	−**29.5 (−46.8 to −12.1**)

Coefficients with p≤0.05 are shown in bold.

GDP, gross domestic product.

Our analysis suggested that increases in the price differentials, representing opportunities for smokers to switch to less expensive cigarettes, may be associated with greater consumption in the same year (6.4 cigarettes per capita for an increase of 10% in price differential, −40.0 to 52.6) and are associated with greater consumption in the following year (67.6 cigarettes per capita, 25.8 to 109.5).

Results from models adjusted for GDP were similar, with price differentials associated with increased consumption of cigarettes in the following year (67.4 cigarettes per capita for an increase of 10% in price differential (25.6 to 109.2) (online supplementary appendix table 1). Models not controlling for time lags were also similar, although associations were concentrated within the year. For example, in these models, price differentials were associated with increased cigarette consumption in the same year (52.1 cigarettes per capita for an increase of 10% in differential (12.2 to 91.9) (online supplementary appendix table 2).

10.1136/tobaccocontrol-2019-055299.supp1 Supplementary data

## Discussion

These findings suggest that differential cigarette pricing strategies may be undermining efforts to control tobacco use. The focus on Europe here is relevant, as Europe generally has higher levels of tobacco tax and prices than many other jurisdictions, including many LMICs, where the majority of the health burden lies.

This analysis adds to a relatively sparse literature on the links between cigarette pricing strategies and consumption using data from 23 European countries. We have relied on a recently released dataset, the ICCD, produced from systematically collected data, which gave greater weight to official sources of data. ICCD also produced their estimates of cigarette consumption by aggregating data on trade, sales and production, rather than survey data alone.^12^ We used price differentials as the exposure in this analysis, as this is a direct marker of opportunities for smokers to switch to less expensive cigarettes. Additionally controlling for GDP, and not including time lags in our models, produced similar results, which increases confidence in these findings.

Some limitations of this study should be considered. We used an ecological study design, which means that we cannot draw conclusions at the individual level. Additionally, we had no price data for five of the 28 EU countries, and thus these findings cannot be ascribed to the whole EU population. Additionally, we were unable to ascertain the exact market share using our price data, although this was above 90% for every country and year. We were unable to assess individual-level relationships between average price, price differentials and consumption, and this remains a fruitful avenue for future research. Finally, our analysis focused only on cigarettes, and not on roll-your-own tobacco, which is known to be less expensive than cigarettes, and may provide an alternative for smokers to maintain tobacco use.

Tobacco taxation is widely considered to be the most effective strategy for tobacco control. Recent evidence has highlighted the techniques which are used by the tobacco industry to prevent the effectiveness of such efforts, indicating that innovations in policy design may be required to make the best use of tax rises. This may include the introduction of a floor, or minimum prices, which would be more difficult for the tobacco industry to combat and would explicitly recognise the concern that smokers move to budget cigarettes. Evidence suggests that in the absence of budget cigarettes, smokers respond to price rises with greater efforts to stop smoking, which would be especially beneficial in tackling inequalities in tobacco use and associated health outcomes.^17 18^

Higher prices of cigarettes are known to be effective in reducing use which can be particularly beneficial for poorer communities, and also has a role in supporting sustainable development goals on non-communicable disease and reducing global inequality.^19^ Higher prices may improve health for poorer individuals, but this relies on people quitting or reducing use, and so dedicating specific revenue from taxes to assist low-income groups quit may be warranted.^20^ Evidence from Europe suggests that greater tax rises would reap more tobacco tax revenue to pay for further tobacco control efforts and that these would be larger in historically more deprived Eastern European countries.^21^ Achieving the aim of eliminating tobacco use will require enhanced adherence to all aspects of the FCTC, including tackling budget cigarettes through article 6. Future research should investigate the response of the tobacco industry to tax and price policies in other settings, including those with less stringent adherence to the FCTC.

## Conclusions

This analysis of data on cigarette prices, price differentials and cigarette consumption within Europe suggests that differential cigarette pricing strategies may undermine progress in tobacco control. Further research is needed on policy measures to deal with strategies employed by the tobacco industry to ensure the availability of budget cigarettes, especially in growth markets such as LMICs.

**What this paper adds**

**What is already known on this topic**

- Raising prices of tobacco is one of the most effective ways of reducing tobacco use
- The tobacco industry uses a range of methods to keep tobacco relatively cheap, including promoting budget cigarettes
- There is limited research on the issue of the availability of budget cigarettes and consumption.

**What this study adds**

- Using data from 23 countries in Europe this study finds increased availability of budget cigarettes to be linked to consumption in the following year
