# Supplementary material for: Associations between cigarette prices and consumption in Europe 2004–2014
Source: Tob Control. 2020 Jun 16;30(1):111–3. doi: 10.1136/tobaccocontrol-2019-055299 (PMC7803893; doi:10.1136/tobaccocontrol-2019-055299)
Supplement: Supplementary data [file tobaccocontrol-2019-055299supp001.pdf]

Appendix

Appendix table 1: Associations between median cigarette prices and price differentials with population-weighted tobacco consumption in 23 European countries 2004 – 2014 (additionally controlling for GDP)

	β (95% CI)
Median cigarette price (per €1 increase)	
Within year	-103.3 (-220.5 to 13.9)
1-year lag	-41.0 (-159.1 to 77.2)
Price differential between median and minimum cigarette price (per 10% increase)	
Within year	6.0 (-40.0 to 52.2)
1-year lag	**67.4 (25.6 to 109.2)**
GDP per capita (per €1000 increase)	-5.3 (-24.5 to 13.9)
Unemployment (per 1% increase)	**-43.5 (-56.6 to -30.4)**
Time (annual)	**-31.0 (-49.1 to -12.8)**

Coefficients with p≤0.05 are shown in bold

Appendix table 2: Associations between median cigarette prices and price differentials with population-weighted tobacco consumption in 23 European countries 2004 – 2014 (without controlling for time lags)

	β (95% CI)
Median cigarette price (per €1 increase)	
Within year	**-168.4 (237.0 to 99.9)**
1-year lag	-
Price differential between median and minimum cigarette price (per 10% increase)	
Within year	**52.1 (12.2 to 91.9)**
1-year lag	-
Unemployment (per 1% increase)	-37.7 (-47.0 to 16.5)
Time (annual)	**-31.7 (-47.0 to -16.5)**

Coefficients with p≤0.05 are shown in bold
